# Heart rate variability and autonomic function in health professions students: Associations with academic stress and lifestyle factors

**DOI:** 10.14814/phy2.71012

**Published:** 2026-07-06

**Authors:** Azza Alawad, Tarig Merghani, Nishaat Chowdhury, Hibat Alla Hisham

**Affiliations:** ^1^ Faculty of Medicine Al‐Neelain University Khartoum Sudan; ^2^ RAK College of Medical Sciences RAK Medical and Health Sciences University Ras Al‐Khaimah UAE

**Keywords:** autonomic nervous system, heart rate variability, medical students, obesity, sleep, stress

## Abstract

Heart rate variability (HRV) is a non‐invasive measure of autonomic nervous system function and a sensitive marker of physiological stress. Health professions students are exposed to severe academic stress and multiple lifestyle‐related factors that may influence autonomic regulation. This study aimed to evaluate HRV profiles in health professions students and examine associations with academic stress and lifestyle factors. A cross‐sectional study was conducted among 237 students (81.4% females). HRV was assessed under standardized resting conditions using time‐domain, frequency‐domain, and Poincaré plot‐derived geometrical indices. Autonomic status was classified as normal autonomic function (NAF), low HRV (LHRV), parasympathetic dominance (PD), or sympathetic dominance (SD). Associations with body mass index, smoking, physical activity, sleep quality, and perceived stress were analyzed using chi‐square and correlation tests. Normal autonomic function was observed in 65.0% of students, while 35.0% exhibited autonomic imbalance (16.9% PD, 16.9% SD, and 1.3% LHRV). No significant associations were found with lifestyle factors (*p* > 0.05). Perceived academic stress was significantly associated with autonomic imbalance among females (*p* = 0.013). In conclusion, most students demonstrated preserved autonomic function, although approximately one‐third showed autonomic imbalance. Academic stress was significantly associated with autonomic function, particularly among female students.

## 1 INTRODUCTION

Heart rate variability (HRV), defined as the beat‐to‐beat variation in the duration of the cardiac cycle, reflects the dynamic relationship between the sympathetic and parasympathetic divisions of the autonomic nervous system (Shaffer & Ginsberg, 2017). A healthy cardiovascular system is characterized by greater variability, indicating compliant and adaptive autonomic regulation, whereas autonomic imbalance, which is associated with physiological stress, fatigue, and increased cardiovascular risk, is characterized by reduced variability (da Estrela et al., 2021; Escorihuela et al., 2020; Immanuel et al., 2023).

In recent years, HRV has emerged as a valuable non‐invasive indicator of psychological stress, mental health, and overall physiological resilience (da Estrela et al., 2021; Guichard et al., 2024; Immanuel et al., 2023). Reduced HRV has been linked to anxiety, burnout, metabolic disturbances, and cardiovascular disease, while balanced autonomic activity reflects effective stress adaptation and homeostatic stability (da Estrela et al., 2021; Guichard et al., 2024; Immanuel et al., 2023; Lehrer & Gevirtz, 2014; Wang et al., 2026). The alterations in autonomic function highlight the value of HRV as a non‐invasive measure of autonomic regulation in both health and disease (Guichard et al., 2024; Shaffer & Ginsberg, 2017).

Heart rate variability (HRV) indices reflect different aspects of autonomic regulation and are broadly categorized into time‐domain, frequency‐domain, and geometrical measures. Time‐domain indices, such as the standard deviation of normal‐to‐normal intervals (SDNN) and the root mean square of successive differences (RMSSD), provide simple measures of overall variability and are strongly influenced by parasympathetic activity (Kim et al., 2018; Shaffer & Ginsberg, 2017). Frequency‐domain measures, including low‐frequency (LF) and high‐frequency (HF) power, assess the distribution of heart rate oscillations across specific frequency bands and can be used to explore autonomic modulation (Kim et al., 2018; Shaffer & Ginsberg, 2017). Geometrical indices, such as SD1 and SD2 derived from Poincaré plots, capture the complexity and dynamic behavior of cardiac autonomic regulation, offering additional insight beyond traditional linear metrics. Collectively, these indices provide complementary information about autonomic function, with higher HRV generally reflecting greater physiological adaptability and resilience (Kim et al., 2018; Shaffer & Ginsberg, 2017). Therefore, HRV analysis serves as a valuable non‐invasive tool for assessing cardiovascular and autonomic health.

In the context of stress, it is found that psychological or cognitive stress is associated with reduced HRV, primarily due to decreased vagal activity and increased sympathetic dominance (Guichard et al., 2024; Immanuel et al., 2023; Kim et al., 2018; Lehrer & Gevirtz, 2014; Wang et al., 2026). It is worth noting that health professions students are exposed to significant academic demands, time tension, and exam‐related stress. These challenges are often accompanied by adverse lifestyle patterns, including reduced physical activity, irregular sleep, smoking, and weight gain (Mahfouz et al., 2024; Palijo et al., 2025). Previous studies have demonstrated that obesity and smoking are associated with reduced HRV, reflecting increased sympathetic activity and diminished vagal modulation, whereas regular physical activity enhances parasympathetic tone and improves autonomic balance (Bezerra, 2025; Strüven et al., 2021; Tiwari et al., 2021; Wong & Figueroa, 2021). Sleep quality is also a key determinant of autonomic regulation, with poor sleep associated with sympathetic predominance and reduced HRV (Bezerra, 2025).

Despite the accumulating evidence, the relative contributions of academic stress and lifestyle factors to autonomic function in healthy young adults remain unclear. Furthermore, data from the Middle East and from health professions students are limited. A clearer understanding of these relationships is essential for developing targeted interventions to improve student well‐being and reduce long‐term cardiovascular risk. Therefore, this study aimed to identify HRV profiles among health professions students, examine the associations between autonomic function and academic stress and lifestyle factors, and evaluate the effects of obesity, smoking, and physical activity on HRV.

## 2 METHODS

### 2.1 Study design and setting

This cross‐sectional study was conducted in the Physiology laboratory at the College of Medicine, Ras Al Khaimah Medical and Health Sciences University, United Arab Emirates, between December 2024 and June 2025. The study targeted undergraduate students enrolled in various health professions programs.

Ethical approval with the number “RAKMHSU‐HEC‐131‐UG‐M” was obtained from the Human Ethics Committee of Ras Al Khaimah Medical and Health Sciences University. The study was conducted in accordance with the principles outlined in the Declaration of Helsinki, and all participants provided written informed consent before participating in the study.

### 2.2 Study population and sampling

A total of 237 students aged between 18 and 30 years were randomly selected from different academic years and disciplines to ensure representation across the student population. Students were informed about the study through class announcements within their respective health professions programs. Eligibility was restricted to currently enrolled students who provided written informed consent. Individuals with known cardiovascular disease, diabetes mellitus, thyroid disorders, or other conditions known to affect autonomic function were excluded. Additional exclusion criteria included the use of medications known to affect heart rate variability, such as beta‐blockers or antidepressants, and the use of illicit substances.

### 2.3 Data collection and lifestyle assessment

Data was collected during periods of regular teaching activities and outside major examination periods to minimize the influence of acute examination‐related stress on HRV measurements and to assess autonomic function under typical academic conditions. A structured questionnaire was utilized to collect information about demographic characteristics, information on smoking status, physical activity patterns, and perceived academic stress. Sleep quality was assessed using the Pittsburgh Sleep Quality Index, a validated instrument that evaluates multiple dimensions of sleep over the preceding month (Buysse et al., 1989). A global score greater than five was used to indicate poor sleep quality. Body weight and height were measured using calibrated equipment, and body mass index was calculated as weight in kilograms divided by the square of height in meters (kg/m^2^). Participants were subsequently categorized as normal weight (18.5–24.9 kg/m^2^), overweight (25.0–29.9 kg/m^2^), or obese (≥30.0 kg/m^2^) according to the World Health Organization classification (World Health Organization, 2000).

### 2.4 Heart rate variability measurement

Heart rate variability was assessed under standardized resting conditions using a four‐channel PowerLab data acquisition system (ADInstruments, Australia) integrated with LabChart software. Cardiac electrical activity was recorded using a surface electrocardiogram (ECG) with appropriately placed limb electrodes to ensure optimal signal quality. All recordings were performed in a quiet, temperature‐controlled environment. Preparations before measurement included avoidance of heavy meals for at least 2 h and stopping caffeine, smoking, and hard physical activity for at least 24 h prior to testing. Patients were allowed to rest for about 5–10 min to ensure cardiovascular stabilization. Heart rate variability was recorded continuously for 3–5 min during rest and spontaneous breathing. Beat‐to‐beat R–R intervals were obtained and carefully inspected, and only normal‐to‐normal intervals were included in the analysis after exclusion of artifacts. Parameters obtained included time‐domain indices (standard deviation of normal‐to‐normal intervals or SDNN and root mean square of successive differences or RMSSD), frequency‐domain measures (low‐frequency or LF and high‐frequency or HF power and their ratio or LF/HF), and Poincaré plot‐derived geometrical indices (SD1 and SD2) to further characterize autonomic dynamics. These parameters are widely used, physiologically interpretable, and recommended by established HRV guidelines for the assessment of autonomic function (Shaffer & Ginsberg, 2017; Electrophysiology TF, 1996); however, additional advanced HRV measures, such as entropy‐based indices, symbolic dynamics, predictability measures, and heart rate asymmetry (Maestri et al., 2007), were not included in the present study.

### 2.5 Autonomic function classification

Since there is no universally accepted classification algorithm for categorization of autonomic function using short‐term resting HRV recordings, we classified autonomic function based on established physiological interpretations of HRV metrics reported in the Task Force of the European Society of Cardiology and the North American Society of Pacing and Electrophysiology and subsequent reviews of HRV methodology (Shaffer & Ginsberg, 2017; Electrophysiology TF, 1996). The classification utilized a collective and predominant pattern across multiple HRV parameters rather than relying on a single indicator. Parameters included time‐domain indices (SDNN, RMSSD, and pNN50), frequency‐domain indices (LF, HF, and LF/HF ratio), and Poincaré plot‐derived indices (SD1 and SD2). Published normal values for short‐term HRV recordings in healthy adults, including SDNN approximately 50 ± 16 ms, RMSSD approximately 42 ± 15 ms, LF power approximately 519 ± 291 ms^2^, HF power approximately 657 ± 777 ms^2^, LF normalized units approximately 52 ± 10, HF normalized units approximately 40 ± 10, and LF/HF ratio approximately 2.8 ± 2.6 were used as reference values during interpretation (Shaffer & Ginsberg, 2017; Electrophysiology TF, 1996). The participants were classified as having normal autonomic function when HRV parameters demonstrated balanced autonomic modulation without evidence of marked sympathetic or parasympathetic predominance. Parasympathetic dominance was characterized by relatively higher vagally mediated indices, including RMSSD, pNN50, HF power, and SD1, together with lower LF/HF ratios. Sympathetic dominance was characterized by relatively lower vagally mediated indices, higher LF/HF ratios, and reduced SD1 values. Participants demonstrating generalized reductions in overall HRV indices, including SDNN, RMSSD, HF power, LF power, SD1, and SD2, were classified as having low HRV (Shaffer & Ginsberg, 2017; Electrophysiology TF, 1996).

### 2.6 Statistical analysis

Statistical analysis was performed using the Statistical Package for the Social Sciences software (version 26.0; IBM Corp., USA). Continuous variables were expressed as mean and standard deviation, whereas categorical variables were presented as frequencies and percentages. Associations between categorical variables were evaluated using the chi‐squared test or Fisher's exact test, as appropriate, and relationships between continuous variables were examined using Pearson correlation analysis. Effect sizes were estimated using Cramer's V. A *p*‐value of <0.05 was considered statistically significant.

## 3 RESULTS

Table 1 shows the baseline characteristics of the study participants. A total of 237 undergraduate health professions students (81.4% females) took part in the study. Most students had a normal body mass index (63.3%), while 26.6% were overweight and 10.1% were obese. Poor sleep quality was reported by 74.3% of participants. The majority were non‐smokers (88.6%), and a considerable proportion of students (60.3%) reported high levels of perceived academic stress.

**TABLE 1:** Baseline characteristics of participants.

Variable	Category	*n* (%)
Gender	Female	193 (81.4)
	Male	44 (18.6)
BMI	Normal	150 (63.3)
	Overweight	63 (26.6)
	Obese	24 (10.1)
Sleep quality	Good	61 (25.7)
	Poor	176 (74.3)
Smoking status	Non‐smoker	210 (88.6)
	Active smoker	27 (11.4)
Perceived academic stress	High (daily/weekly)	143 (60.3)
	Low (monthly/rarely/never)	94 (39.7)

Table 2 presents the descriptive statistics of the data. The mean age of participants was 20.1 ± 1.9 years, with a mean body mass index of 23.6 ± 4.3 kg/m^2^ and a mean sleep index score of 7.5 ± 2.9. HRV analysis demonstrated overall preserved autonomic function, with mean SDNN and RMSSD values of 62.1 ± 30.8 ms and 56.9 ± 33.2 ms, respectively. Other HRV indices, including pNN50, frequency‐domain measures, and Poincaré plot‐derived geometrical parameters, showed considerable variability, indicating heterogeneity in autonomic regulation within the study population.

**TABLE 2:** Baseline characteristics and heart rate variability parameters of the study population (*n* = 237).

Parameter	Mean ± SD
Age	20.1 ± 1.9
Body mass index	23.6 ± 4.3
Sleep index	7.5 ± 2.9
SDNN (ms)	62.1 ± 30.8
RMSSD (ms)	56.9 ± 33.2
pNN50 (%)	23.8 ± 22.3
LF (nu)	34.2 ± 23.9
HF (nu)	35.9 ± 21.8
LF/HF ratio	1.4 ± 1.5
SD1 (ms)	39.9 ± 39.4
SD2 (ms)	74.8 ± 39.8

Table 3 shows autonomic function classification based on the integrated HRV classification. Normal autonomic function was identified in 65.0% of participants. A considerable proportion demonstrated autonomic imbalance with parasympathetic dominance (16.9%), sympathetic dominance (16.9%), and low HRV (1.3%).

**TABLE 3:** Autonomic function classification.

Category	*n* (%)
Normal autonomic balance	154 (65.0)
Parasympathetic dominance	40 (16.9)
Sympathetic dominance	40 (16.9)
Low HRV	3 (1.3)

Table 4 shows insignificant statistical associations between autonomic function classification and body mass index categories, smoking status, physical activity, and sleep quality (*p* > 0.05). Table 5 demonstrated a significant association between perceived academic stress and autonomic function among female students (Fisher's exact test, *p* = 0.013), with higher stress levels being associated with deviations from normal autonomic balance. The strength of the association was moderate (Cramer's V = 0.201). Although the association was not statistically significant among males (Fisher's exact test, *p* = 0.074), the moderate effect size (Cramer's V = 0.299) suggests that a relationship should be explored further in studies with larger sample sizes.

**TABLE 4:** Association between lifestyle factors and autonomic function.

Variable	*χ* ^2^	*p‐*value
BMI	8.637	0.471
Smoking	1.09	0.982
Exercise	3.09	0.961
Sleep quality	1.77	0.622

**TABLE 5:** Association between perceived academic stress and autonomic function classification by gender.

Gender	Stress level	Low HRV	Normal	Parasymp. dominance	Sympathetic dominance	Total *n* (%)	*p* value
Female *N* = 193	Daily‐weekly	0 (0.0)	98 (68.5)	19 (13.3)	26 (18.2)	143 (74.1)	0.013
	Monthly	2 (6.1)	22 (66.7)	5 (15.2)	4 (12.1)	33 (17.1)	
	Never‐rarely	1 (5.9)	7 (41.2)	6 (35.3)	3 (17.6)	17 (8.8)	
Male *N* = 44	Daily‐weekly	0 (0.0)	11 (52.4)	4 (19.0)	6 (28.6)	21 (47.7)	0.074
	Monthly	0 (0.0)	3 (42.9)	3 (42.9)	1 (14.3)	7 (15.9)	
	Never‐ rarely	0 (0.0)	13 (81.3)	3 (18.7)	0 (0.0)	16 (36.4)	

*Note*: Fisher's exact test was used for statistical analysis. The strength of association was assessed using Cramer's V (female: 0.201; male: 0.299).

Correlation analysis in Table 6 reveals no significant associations between body mass index or sleep quality scores and any heart rate variability indices. The strong positive correlations among time‐domain and Poincaré plot‐derived geometrical HRV indices, specifically between SDNN, RMSSD, pNN50, SD1, and SD2, indicate a high degree of internal consistency and reflect shared parasympathetic modulation. Most of these significant correlations remained significant following Benjamini–Hochberg false discovery rate correction for multiple comparisons while only the correlations between RMSSD and LF (*p* = 0.026) and between SD1 and LF (*p* = 0.034) did not meet the adjusted significance threshold. The correlation between RMSSD and SD1 confirms that both indices represent the same aspect of short‐term vagal activity. Overall, these findings support the strength of time‐domain and Poincaré plot‐derived geometrical indices as indicators of parasympathetic function.

**TABLE 6:** Correlation matrix of body mass index, sleep quality, and heart rate variability indices (*n* = 237).

Parameter	BMI	Sleep score	SDNN	RMSSD	pNN50	LF	HF	LF/ HF	SD1	SD2
BMI	1	−0.004	−0.054	−0.088	−0.038	−0.032	−0.042	0.081	−0.087	−0.015
		0.947	0.406	0.177	0.560	0.627	0.518	0.212	0.181	0.820
Sleep score	−0.004	1	0.067	0.064	0.026	−0.111	−0.048	0.004	0.059	0.063
	0.947		0.301	0.328	0.688	0.088	0.463	0.957	0.362	0.333
SDNN	−0.054	0.067	1	0.920*	0.807*	−0.161*	−0.086	−0.243*	0.921*	0.903*
	0.406	0.301		0.000	0.000	0.013	0.184	0.000	0.000	0.000
RMSSD	−0.088	0.064	0.920*	1	0.839*	−0.144*	−0.105	−0.310*	0.999*	0.683*
	0.177	0.328	0.000		0.000	0.026	0.105	0.000	0.000	0.000
pNN50	−0.038	0.026	0.807*	0.839*	1	−0.170*	−0.163*	−0.356*	0.840*	0.648*
	0.560	0.688	0.000	0.000		0.009	0.012	0.000	0.000	0.000
LF	−0.032	−0.111	−0.161*	−0.144*	−0.170*	1	0.113	0.031	−0.138*	−0.157*
	0.627	0.088	0.013	0.026	0.009		0.083	0.631	0.034	0.015
HF	−0.042	−0.048	−0.086	−0.105	−0.163*	0.113	1	0.010	−0.106	−0.083
	0.518	0.463	0.184	0.105	0.012	0.083		0.884	0.105	0.205
LF/ HF	0.081	0.004	−0.243*	−0.310*	−0.356*	0.031	0.010	1	−0.309*	−0.165*
	0.212	0.957	0.000	0.000	0.000	0.631	0.884		0.000	0.011
SD1	−0.087	0.059	0.921*	0.999*	0.840*	−0.138*	−0.106	−0.309*	1	0.683*
	0.181	0.362	0.000	0.000	0.000	0.034	0.105	0.000		0.000
SD2	−0.015	0.063	0.903*	0.683*	0.648*	−0.157*	−0.083	−0.165*	0.683*	1
	0.820	0.333	0.000	0.000	0.000	0.015	0.205	0.011	0.000	

* *p* < 0.05.

## 4 DISCUSSION

This study provides an overview of autonomic function among health professions students using multi‐domain heart rate variability analysis. The findings demonstrate that most participants exhibited preserved autonomic regulation, as reflected by relatively normal HRV indices. This observation is consistent with the generally favorable cardiovascular profile expected in young, healthy adults. However, approximately one‐third of students demonstrated patterns of autonomic imbalance, highlighting the presence of measurable differences in autonomic regulation among a substantial proportion of the study population.

Our study showed the absence of significant associations between HRV indices and lifestyle‐related factors such as body mass index, smoking status, physical activity, and sleep quality. While previous studies have reported reduced HRV in individuals with obesity and sedentary behavior, these associations are more consistently observed in older people or in those with prolonged exposure to cardiometabolic risk factors (da Estrela et al., 2021; Koenig et al., 2014; Mahfouz et al., 2024; Thayer et al., 2010). It is most likely that autonomic regulation in young adults remains relatively intact until the cumulative effects of risk factors become sufficient to produce measurable change in HRV. For instance, obesity has been associated with reduced parasympathetic activity and increased sympathetic tone in clinical populations, particularly in individuals with severe or long‐standing metabolic dysfunction (Tentolouris et al., 2008). The lack of such associations in the present study may be related to the relatively young age of the participants and the absence of overt metabolic disease.

The absence of a significant relationship between sleep quality and HRV in our study contradicts previous studies linking poor sleep to sympathetic predominance and reduced vagal tone (da Estrela et al., 2021). However, the high prevalence of poor sleep quality in this cohort may have limited the ability to detect meaningful differences between groups. In addition, reliance on self‐reported sleep measures rather than objective methods like polysomnography may not fully capture physiological sleep disturbances.

A key finding of this study is the association between perceived academic stress and autonomic imbalance, particularly among female students. This finding indicates that psychological stress is associated with autonomic regulation, most likely through modulation of vagal tone. Previous studies confirmed the association between stress, whether acute or chronic, and reduced HRV, reflecting diminished parasympathetic activity and impaired autonomic flexibility (da Estrela et al., 2021; Guichard et al., 2024; Immanuel et al., 2023; Lehrer & Gevirtz, 2014; Wang et al., 2026). The observed sex‐specific association in this study may be explained by variation in stress perception, reaction to stress, circulating hormones and coping strategies between males and females (Kudielka & Kirschbaum, 2005). However, despite the absence of statistical significance among males, the observed large effect size indicates that a significant association can be explored further in studies with greater male representation.

The finding that both sympathetic and parasympathetic dominance patterns are found among the undergraduate students reflects the complexity of autonomic responses to psychological stimuli. Sympathetic predominance is typically associated with increased arousal, enhanced cardiovascular responses, and reduced vagal modulation, whereas parasympathetic dominance may reflect compensatory or recovery mechanisms following stress exposure. These patterns are not necessarily indicative of abnormality but may represent dynamic and adaptive responses within the autonomic nervous system. However, chronic imbalance may increase susceptibility to mental health conditions and impair stress resilience.

The strong correlations observed among time‐domain and Poincaré plot‐derived geometrical HRV indices, particularly between RMSSD and SD1, confirm the internal consistency of parameters reflecting parasympathetic modulation. These findings support the robustness of the HRV measurements in this study and are consistent with established physiological relationships between linear and Poincaré plot‐derived geometrical indices of autonomic function (Shaffer & Ginsberg, 2017). However, it is worth noting that although short‐term HRV recordings of 3–5 min are widely used and reported in the literature, frequency‐domain measures, including LF power and the LF/HF ratio, may show greater variability due to short‐term physiological fluctuations and spontaneous breathing. Therefore, their interpretation requires careful consideration. Furthermore, the present study focused on the widely used conventional HRV parameters that are commonly reported in previous studies (Shaffer & Ginsberg, 2017; Electrophysiology TF, 1996; Thayer et al., 2010; Wong & Figueroa, 2021), while additional advanced HRV measures were not evaluated. In addition, the classification of autonomic status in the present study is not a definitive clinical classification. Although HRV is a recognized non‐invasive measure of autonomic function, there is no universally accepted classification system for categorization of autonomic function into sympathetic dominance, parasympathetic dominance, normal autonomic balance, or low HRV based on values of HRV indices. Therefore, the categories used in this study should be interpreted as descriptive physiological profiles rather than validated clinical diagnoses. However, the classification was based on the integrated profile of multiple HRV indices rather than a single parameter, which allows better interpretation. Future studies should explore the development of standardized algorithms for autonomic profiling using HRV data.

## 5 LIMITATIONS

This study has several limitations that should be considered when interpreting the findings. Cross‐sectional design limits the ability to determine causes or describe long‐term influences on autonomic function. Conducting the study in a single institution may restrict the generalizability of the results to other populations. The use of self‐reported measures for assessment of physical activity, smoking, and perceived stress is likely to introduce recall and report bias. Although HRV assessments were performed during non‐examination periods, seasonal influences on psychological well‐being and sleep behavior were not evaluated. The predominance of female participants limited the statistical power of analyses among males, and menstrual history was not recorded. In addition, habitual caffeine consumption was not assessed beyond the pre‐test restriction period. In addition, the short‐term recordings of HRV for only 3–5 min may show greater variability of the frequency‐domain parameters (LF power and the LF/HF ratio) because of the physiological fluctuation of autonomic discharge during spontaneous breathing. Added to that, although HRV during resting conditions is a well‐established measure of basal autonomic regulation, it does not fully capture autonomic responsiveness to external stimuli such as postural change, controlled breathing, or exercise. Future studies combining HRV with dynamic autonomic function tests may provide further information about autonomic adaptability. Another limitation of this study is the categorization of autonomic function based on descriptive physiological patterns rather than definitive diagnostic classifications due to the lack of universally accepted classification that is based on short‐term resting HRV recordings. In addition, although the utilized conventional HRV indices are widely used and supported by HRV guidelines, several advanced HRV measures such as entropy analysis, symbolic dynamics, predictability indices, and heart rate asymmetry were not assessed. These parameters may contribute to a more comprehensive assessment of autonomic function. Finally, multivariable analyses are required to determine the relative contributions of stress, sleep quality, and other lifestyle factors.

## 6 CONCLUSION

In conclusion, most health professions students in this study demonstrated preserved autonomic function; however, a substantial proportion exhibited patterns of autonomic imbalance, indicating the need for greater attention to autonomic health and well‐being among students in highly demanding academic environments. Academic stress showed significant association with autonomic function, particularly among female students, whereas lifestyle factors showed no measurable impact in this young cohort. These findings highlight the dominant role of psychological stress in modulating autonomic function in early adulthood and emphasize the need for targeted interventions aimed at improving stress management and overall well‐being among students. Our findings suggest that heart rate variability is a valuable, non‐invasive tool for assessing autonomic function and exploring factors associated with student well‐being and academic stress. Future studies employing longitudinal designs and objective measures of lifestyle factors are highly recommended to further explain the impact of these factors on autonomic function.

## AUTHOR CONTRIBUTIONS

**Azza Alawad:** Conceptualization; data curation; formal analysis; investigation; methodology. **Tarig Merghani:** Conceptualization; formal analysis; investigation; methodology; resources; software; supervision. **Nishaat Chowdhury:** Conceptualization; data curation; formal analysis; investigation; methodology; project administration. **Hibat Alla Hisham:** Conceptualization; data curation; formal analysis; investigation; methodology; project administration.

## FUNDING INFORMATION

The authors have nothing to report.

## CONFLICT OF INTEREST STATEMENT

The authors declare no conflicts of interest.

## Data Availability

The data that support the findings of this study are available from the corresponding author upon reasonable request.
